# What can we learn from precise reporting of residual disease after various types of cholesteatoma surgery using STAM areas?

**DOI:** 10.1007/s00405-025-09579-3

**Published:** 2025-09-23

**Authors:** Maura C. Eggink, Maarten J. F. de Wolf, Fenna A. Ebbens, Frederik G. Dikkers, Erik van Spronsen

**Affiliations:** Department of Otorhinolaryngology, Amsterdam UMC Location University of Amsterdam, Meibergdreef 9, 1105 AZ Amsterdam, the Netherlands

**Keywords:** Cholesteatoma surgery, Residual disease, STAM areas, Obliteration

## Abstract

**Purpose:**

1. To evaluate current reporting of residual cholesteatoma localisation. 2. To assess prevalent localisations of residual disease following various types of cholesteatoma surgery in our population.

**Methods:**

1. Review of the literature on reported localisation of residual disease following cholesteatoma surgery. 2. Retrospective chart review of patients undergoing cholesteatoma surgery in a tertiary referral centre with a minimum follow-up of two years. Localisation of residual cholesteatoma was noted according to the STAM areas and compared to initial cholesteatoma. Overlapping localisations and multiple pearls were specified.

**Results:**

Overall residual disease rate of 14.4% in 1084 surgeries was similar to the pooled data from the literature. In our population, residual disease was most prevalent in A (attic), followed by T (tympanic cavity). The risk of residual disease in M (mastoid) was low. Surgery type influenced the overall risk of residual disease per localisation, as well as the proportion of affected areas. Obliteration reduced the risk of residual disease. Of the residual pearls, 12% were found remote of initial cholesteatoma localisation.

**Conclusion:**

Surgery type is a determining factor not only in residual disease rate, but also in localisation of residual disease. Both the efficacy and safety of obliteration is underlined. Standardised reporting of residuals utilising STAM areas, including specification of overlapping areas and remote residuals, will facilitate comparative research on surgical factors influencing residual disease, while providing useful insights for otologic surgeons.

**Supplementary Information:**

The online version contains supplementary material available at 10.1007/s00405-025-09579-3.

## Introduction

Residual cholesteatoma is defined as regrowth of disease following incomplete removal of matrix during the initial surgery. One of the determining factors is surgical technique. Historically, canal-wall up procedures (CWU) have been associated with higher residual disease rates than canal-wall down (CWD), 3–40% [1–4] versus 0–30% [3, 5] respectively. In the past decades novel surgical techniques have been introduced to reduce these statistics. Residual disease rates after obliteration have been promising [6–8]. The use of an endoscope, either during total transcanal endoscopic ear surgery or during endoscope-assisted microscopic surgery, has been demonstrated to expose hidden areas of the tympanic cavity with potential benefit in reducing residual disease [9–14]. Despite these evolving surgical techniques, further elimination of residual disease after cholesteatoma surgery remains a challenge.

In addition to intervention-related factors, patient-related factors influencing residual disease have also been identified. Paediatric patients have a higher risk of residual disease, possibly attributable to a more aggressive growth pattern [6, 15–19].

Furthermore, certain localisations of cholesteatoma have been recognized as prone to residual disease. The (anterior) epitympanum is reported as the most common localisation of residual disease, followed by the tympanic cavity, specifically the sinus tympani [2, 9, 10, 20–22]. However, a uniform method of reporting residual disease in the literature is lacking.

The aim of this study was two-fold: 1. to evaluate current literature reporting residual cholesteatoma localisation and 2. to assess prevalent localisations of residual cholesteatoma following various types of cholesteatoma surgery in our population. Residual cholesteatoma localisation was reported using the STAM areas [23], a framework developed by the EAONO/JOS to describe localisation of cholesteatoma in the middle ear and mastoid, later revised by Merkus et al. (S1: anterior epitympanum; S2: sinus tympani; T: tympanic cavity; A: attic and M: mastoid) [23, 24]. Previously, use of the STAM areas was demonstrated to effectively describe cholesteatoma localisation and therefore deemed suitable for description of residual disease localisation [25]. We aim to provide valuable insights for otologic surgeons concerning areas prone to residual disease after specific surgery types, while facilitating comparative research on surgical factors influencing residual disease.

## Materials and methods

### Literature review

A review of the literature was performed on PubMed Central on the 16th of November 2023 using the following search entry: “cholesteatoma”[tiab] AND “residual*”[tiab] AND (“loca*”[tiab] OR “site*”[tiab]). Abstracts were screened and full publications were accessed when inclusion criteria were met. These inclusion criteria were: original articles describing the localisation of residual disease during second-look surgery after both primary and revision cholesteatoma surgery in patients of all ages, noting a minimum follow-up length (FU) or timing of second-look surgery, clear distinction between recurrent and residual disease as well as an adequate description of surgery performed. Studies were excluded when total number of surgeries or number of residual cases could not be derived, when there was no English full-text available or a substantial study population was not met (*n* < 50). Residual disease rate was calculated by extracting incidence of residual disease as proportion of total initial cholesteatoma surgeries performed. Localisation of cholesteatoma residuals were categorised in tympanic cavity; mastoid (including antrum, in line with the EAONO-JOS definitions [23]) and epitympanum based on the available information. In case of multiple pearls per patient or overlapping locations of residual disease, ratios of affected regions were used to calculate residual disease rate per localisation.

### Participants

All patients undergoing both primary and revision cholesteatoma surgery between January 1st 2009 and February 1st 2021 in a tertiary referral hospital were evaluated. Surgeries performed in these patients prior to the inclusion period were also screened. Patients were identified when surgery was performed due to residual disease following previous surgery in our centre, with a minimum FU of two years. Patient age was recorded. Patients were excluded in case of intended macroscopic residue during initial surgery: twice due to drainage of an otogenic abscess, once due to reduced exposition due to profuse bleeding and once due to missing informed consent for mastoidectomy. Another patient was excluded due to doubt of iatrogenic aetiology of cholesteatoma, as previous medical history noted a tympanoplasty without evidence of cholesteatoma.

### Types of surgery

We defined six different approaches (supplementary Table 1): transcanal, including retro-auricular, endaural and total endoscopic (TCA); CWU; CWU with obliteration of the epitympanum and mastoid cavity (CWUO); CWD; CWD with subsequent reconstruction of the posterior canal wall and obliteration of the epitympanum and mastoid cavity (CWD + R) and subtotal petrosectomy with blind sac closure (STP).

### Localisation of disease

Retrospective review of surgical notes was performed. Residual cholesteatoma localisation was noted according to the STAM areas [23]. In case of multiple pearls, localisations were noted separately. When there were doubts regarding the precise localisation, preoperative CTs were consulted if available. Absence of opacification in the area in question was considered free of residual cholesteatoma.

Localisation of residual disease was compared to surgical notes from initial surgery. Residual disease was categorised to be occurring in: A. the previously described STAM areas; B. partially presenting in a novel location, potentially grown from the original location (e.g. initial cholesteatoma in A and T, reoccurring in A and S1) or C. presenting in a novel localisation (e.g. initial cholesteatoma in M and A, residue in S2). The latter category was defined as a “remote residual”: growth of remnant cholesteatoma behind an intact tympanic membrane, in a location different than the originally reported cholesteatoma. This is distinct from a recurrent cholesteatoma; the regrowth of a retraction pocket forming a novel cholesteatoma [24]. A solitary cyst was deemed to be residual disease of that specific localisation, as it could not have grown from an adjacent area.

### Analysis

To calculate residual disease rate, proportionally affected regions were used to account for overlapping areas. Statistical analyses were performed in SPSS 28.0 (Chicago, IL, USA) using occurrence of residual disease (*n* = 156). For specific assessment of affected STAM areas following various types of surgery, multiple pearls per ear were assessed separately (*n* = 206). Normal distribution of data was tested with a Shapiro–Wilk test. Categorical data was tested with a Chi-square test (Fisher’s Exact test where necessary). A *p* < 0.05 was considered statistically significant.

## Results

### Literature review

The literature review yielded 129 English articles. Eleven articles matched the selection criteria (Table 1) [2, 9, 10, 16, 19–21, 26–29]. Timing of second-look and FU varied. All articles used free text to describe residual disease localisation. The majority of studies reported on overlapping localisations, but none reported on all three qualitative aspects: the occurrence of multiple pearls, remote residuals, and overlapping localisations of cholesteatoma. The residual disease rate ranged from 3.3–28.2%, with a pooled rate of 15.3% (*n* = 1715, Fig. 1 and supplementary Fig. 1). In eight out of eleven articles, the most prevalent localisation of residual disease was the tympanic cavity, ranging from 1.6–13.5% with a pooled rate of 7.3%. In seven out of the eleven articles, the mastoid had the lowest chance of residual disease, ranging from 0.4–4.9%, with a pooled rate of 2.1%.

**Table 1:** Characteristics of reviewed studies

*Authors*	*Surgery types*	*Population*	*Timing 2nd-look*	*Follow-up*	*Multiple pearls*	*Overlapping localisations*	*Remote residuals*
Badr-el-Dine et al. [27]	CWU + endoscopy; CWD + endoscopy	NS	9-12mo	NS	No	Yes	No
Gaillardin et al. [2]	TCA with atticotomy ± endoscopy; CWU ± endoscopy	Adults	< 12mo	> 24mo	No	No	Yes
Glikson et al. [28]	TCA + endoscope ± atticotomy; CWU	< 16yrs	NS	> 12mo	No	Yes	No
Gristwood et al. [16]	TCA ± atticotomy; CWU; CWD ± obliteration	All ages	NS	> 60mo	No	No	No
Haginomori et al. [9]	CWD + R (- obliteration)	All ages	± 12mo	NS	Yes	No	No
Hunter et al. [29]	TCA ± endoscope; CWU ± endoscope	≤ 18yrs	NS	> 6mo	No	Yes	No
Iino et al. [19]	CWD + R (- obliteration)	≤ 10yrs	6-18mo	NS	Yes	Yes	No
James et al. [26]	CWU + laser ± endoscope	< 18yrs	± 12mo	> 12mo	No	Yes	No
Sanna et al. [21]	TCA + atticotomy; CWU; CWD; unspecified	All ages	NS	> 12mo	Yes	Yes	No
Rayneau et al. [20]	CWU; CWD + R (+ obliteration)	> 18yrs	NS	> 18mo	No	Yes	No
Yung [10]	TCA + atticotomy + endoscope; CWU + endoscope; CWD(+ R) + endoscope ± obliteration	All ages	12-18mo	> 12mo	No	No	Yes

“Multiple pearls” refers to the specification of residual disease in a single or in multiple pearls within one ear. “Overlapping localisations” refers to specification of residual disease found in overlapping areas. “Remote residuals” refers to the specification of residual disease localisation in regard to the original cholesteatoma. *NS* not specified. *CWU* canal wall up procedure, *CWD* canal wall down procedure, *TCA* transcanal procedure, *CWD* + *R* canal wall down mastoidectomy with subsequent reconstruction of the posterior canal wall. + with,—without

**Fig. 1:**
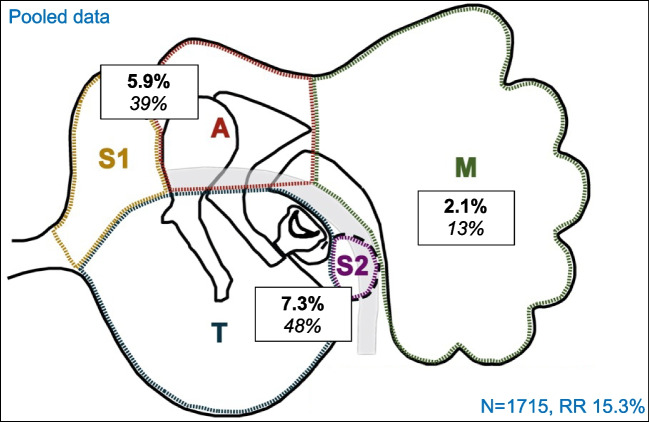
Pooled localisation of residual cholesteatoma in reviewed articles. In bold: residual disease rate in epitympanum (S1 and A), tympanic cavity (T and S2) and mastoid (M), as percentage of all surgeries included. In italic: residual disease per localisation as percentage of all residuals. N: number of surgeries, RR: total residual disease rate

### Participants and overall residual disease rate

In our center, 671 patients underwent 1084 surgical interventions to remove cholesteatoma. Median time between initial surgery and surgery for residual disease was 560 days (IQR 157–964). Median age of patients during initial surgery was 18 years (IQR 4–31). The overall rate of residual disease during follow-up surgery was 14.4% (156/1084). The highest residual disease rate was found following CWU and STP (Table 2). Residual disease rate was significantly influenced by surgery type (*p* < 0.001).

**Table 2:** Residual cholesteatoma rate per surgery type, including residual disease rate per localisation

	*Initial surgery type* N (%)	*Residual disease rate* N (%)	*% Residual disease rate per surgery*	*% Residual disease rate per localisation*				
				*S1*	*S2*	*T*	*A*	*M*
Total	1084 (100)	156 (100)	14.4	1.7	2.5	3.4	4.7	2.0
CWU	459 (42.3)	117 (75.0)	25.5	3.3	4.7	5.2	8.8	3.6
CWUO	361 (33.3)	14 (9.0)	3.9	0.5	0.5	1.6	1.1	0.2
TCA	104 (9.6)	11 (7.0)	10.6	1.1	1.7	2.2	3.9	1.7
CWD	71 (6.5)	8 (5.1)	11.3	0	2.6	4.3	0.9	3.5
CWD + R	59 (5.4)	2 (1.3)	3.4	0	0	2.3	1.1	0
STP	30 (2.8)	4 (2.6)	13.3	2.1	2.1	3.1	3.1	3.1

Residual disease rate: presence of residual disease per surgery type, calculated using proportionally affected areas in case of overlapping regions of multiple pearls. *CWU* canal wall up mastoidectomy; *CWUO* canal wall up mastoidectomy with obliteration of the epitympanum and mastoid cavity; *TCA* transcanal procedure; *CWD* canal wall down mastoidectomy; *CWD* + *R* canal wall down mastoidectomy with subsequent reconstruction of the posterior canal wall and obliteration of the epitympanum and mastoid cavity; *STP* subtotal petrosectomy with blind sac closure. *S1* anterior epitympanum; *S2* sinus tympani; *T* tympanic cavity; *A* epitympanum, *M* mastoid

### Residual cholesteatoma localisation

**Fig. 2:**
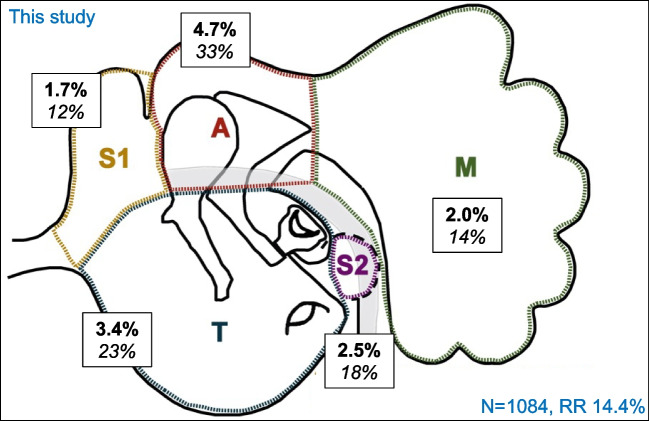
Localisation of residual cholesteatoma per STAM area in this study. In bold residual disease rate per STAM localisation, calculated using proportionally affected areas in case of overlapping regions and multiple pearls (*n* = 156). In italic: residual disease per localisation as percentage of all residuals. N: number of surgeries, RR: total residual disease rate. S1: anterior epitympanum; S2: sinus tympani; T: tympanic cavity; A: epitympanum, M: mastoid

The most common localisation of residual disease was A, followed by T (Table 2 and Fig. 2). The overall risk of residual disease per localisation varied per surgery type (Table 2). The highest risk of residual disease was in A after CWU (8.8%). A low risk of residual disease in the obliterated areas (S1, A and M) was found after CWUO (0.5, 1.1 and 0.2% respectively) and CWD + R (0, 1.1 and 0% respectively). The distribution of residual disease localisation across surgery types was found to be significantly different in T and M (both *p* < 0.05).

**Fig. 3:**
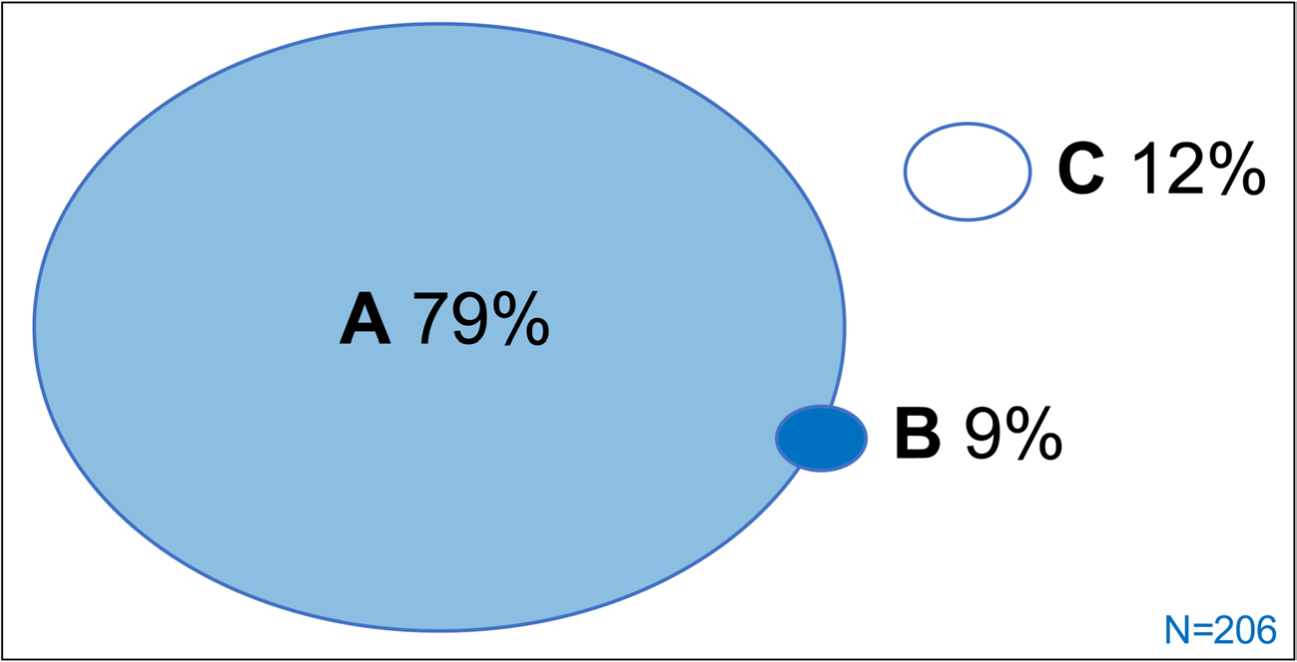
Localisation of residual disease pearls relative to localisation of initial cholesteatoma (*n* = 206). Residual disease pearl localisation in **A**: the previously described STAM areas; **B**: partially presenting in a novel location, potentially grown from the original location; **C**: presenting in a novel localisation, i.e. remote residual. N: number of residual pearls

In 33 surgeries, more than one pearl was found (21.2%), totalling up to 206 residual pearls. Figure 3 illustrates most residual pearls were found in the same localisation as the initial cholesteatoma (group A, 164/206), while 12% were found remote to the initial localisation (group C, 24/206). These remote residuals were found most commonly in S2 and A (both 7/24).

**Fig. 4:**
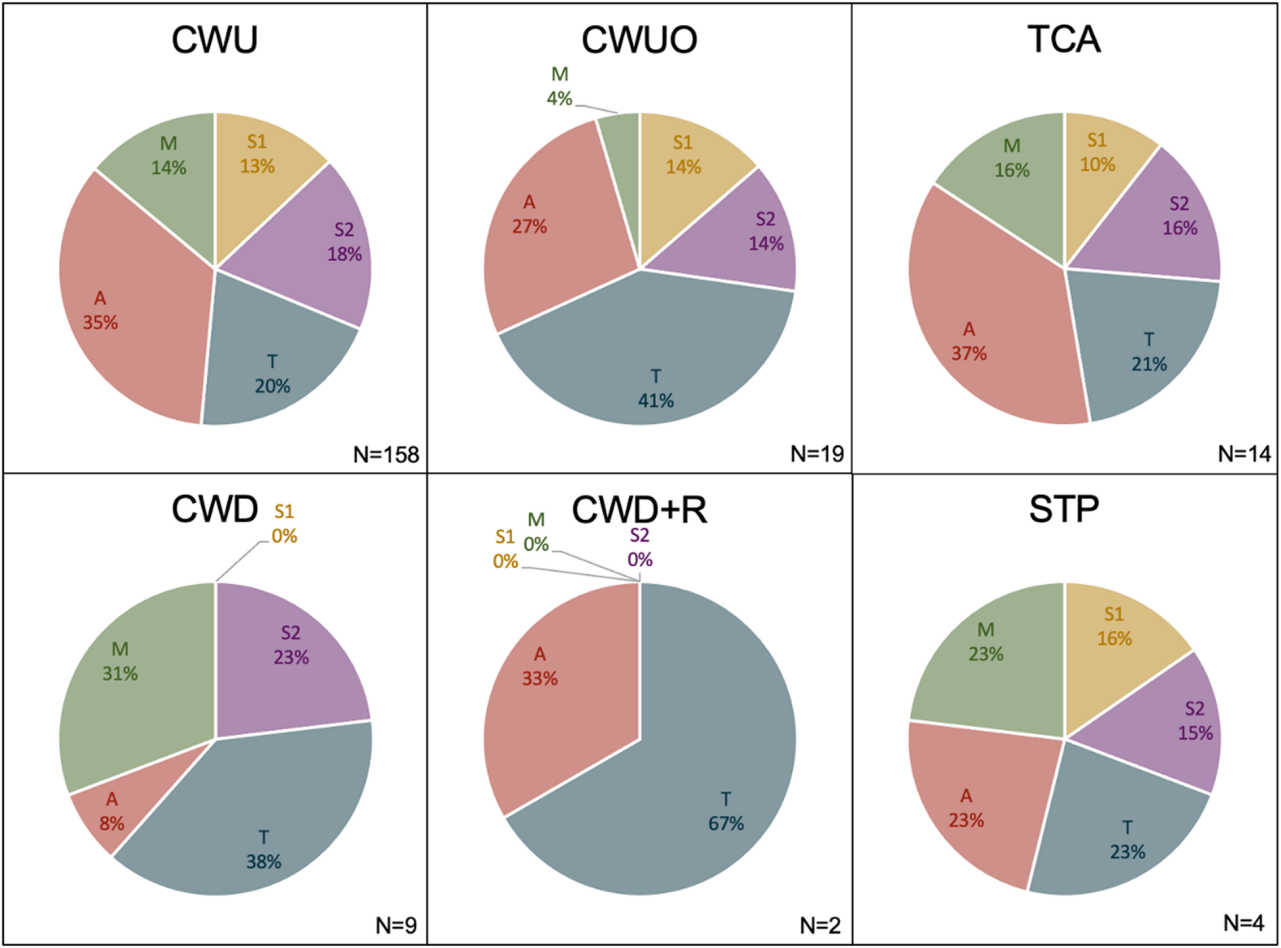
Percentage of STAM areas affected by residual cholesteatoma pearls per surgery type (*n* = 206). CWU: canal wall up mastoidectomy; CWUO: canal wall up mastoidectomy with obliteration of the epitympanum and mastoid cavity; TCA: transcanal procedure; CWD: canal wall down mastoidectomy; CWD + R: canal wall down mastoidectomy with subsequent reconstruction of the posterior canal wall and obliteration of the epitympanum and mastoid cavity; STP: subtotal petrosectomy with blind sac closure. S1: anterior epitympanum; S2: sinus tympani; T: tympanic cavity; A: epitympanum, M: mastoid. N: number of residuals

Of the cholesteatoma pearls, 44 (21.4%) involved more than one STAM area. The proportion of affected areas varied per surgery type (Fig. 4). After CWU and TCA, A was most affected, while T was more affected following CWUO, CWD, CWD + R. After STP, A, T and M were equally affected.

## Discussion

### Residual disease rate

Our overall reported rate of residual disease after cholesteatoma surgery is in line with data provided in the reviewed articles [2, 9, 10, 16, 19–21, 26–29]. This implies our population is an adequate representation of the worldwide cholesteatoma population. The slightly higher pooled residual rate could be influenced by the inclusion of purely paediatric studies, as residual disease has been found to be more common in paediatric patients [6, 16, 30, 31]. This study confirms surgery type as a confounder in residual disease rate [6].

In line with the literature, a substantial drop of residual disease was found when obliteration was performed after CWU (25.5 vs 3.9%) [7, 8, 15, 32]. Despite the maximum exposure achieved during STP, the residual disease rate is the second highest after this specific surgical procedure, following CWU (13.3% and 25.5% respectively). A selection bias for extensive and refractory cholesteatoma is likely. Three out of the four patients receiving STP had undergone previous surgery. In all four cases, adherence of cholesteatoma to soft tissue challenged eradication: disease was dissected off the carotid artery, dura, jugular bulb, internal auditory canal or dehiscent facial nerve.

### Residual disease localisation

Cumulative residual disease rates in S1 and A, as well as in M, are similar to the pooled residual disease rates in the literature (6.4 vs 5.9% and 2.0 vs 2.1% respectively). Cumulative residual disease rate in T and S2 is slightly lower than in the literature (5.9 vs 7.3%). Predominant residual disease localisation in the attic and tympanic cavity could be the direct result of disease traces on a (partially) intact ossicular chain, specifically around the stapes crurae[16]. Also, the often irregular medial surface of the scutum is prone to residual disease [33]. The low risk of residual disease in the mastoid overall, in both our data and the pooled data, underlines the safety of obliteration of the mastoid cavity.

This study demonstrates surgery type not only impacts residual disease rate, but also influences residual disease localisation. As expected, residual disease rate in S1, A, M diminished after obliteration. Obliteration could potentially reduce the viability of remnants of cholesteatoma matrix in the obliterated areas (the “Hinohira effect” [34]). A surgeon might also be less conservative in removal of the scutum prior to obliteration, allowing for increased exposition in the epitympanum [35]. In our center, obliteration of the epitympanum is typically performed after removal of the malleus head and incus. This facilitates further inspection, while reducing the risk of residual disease left behind on an intact ossicular chain. To our surprise, an indirect advantage of obliteration on residual disease seems present in S2 and T. The choice of obliteration could be influenced by a surgeon’s confidence of complete disease eradication in all areas, leading to a slight bias. However, in our data obliteration is performed in 44% of all CWD and CWU procedures. Alternatively, obliteration could have an indirect effect on other areas of the middle ear. It could be hypothesised that obliteration reduces the vascularisation of the epitympanum and mastoid, indirectly reducing the vascularisation of the perimatrix of residual epithelium. This could potentially degrade the viability of epithelial remnants in any area of the middle ear and mastoid.

Removal of the canal wall does seem to have some potential in reducing residual disease in S1 and S2. Despite our overall substantial population size, it is possible the low number of residuals specifically following CWD and CWD + R (*n* = 10) led to an incomplete distribution of residual disease localisations in this subgroup. We agree with other authors that removing the canal wall does provide access to the facial recess, but not to the sinus tympani [2, 10, 36]. Also, overall residual disease rates of CWUO and CWD + R are comparable, suggesting a limited benefit of removing the canal wall. We therefore advocate to maintain the normal anatomy of the ear canal, as this has benefits for acoustic hearing, water tolerance and hygienic care [35]. Due to the heterogeneity of the TCA group, the added benefit of using an endoscope to visualise hidden areas, such as S2, could be not be evaluated.

Only one other study analysed the influence of surgery type on localisation of residual disease, albeit in a smaller population (*n* = 165) [20]. Their study suggests a positive influence of removal of the canal wall and obliteration on both residuals in the mastoid and tympanic cavity.

Our rate of remote residuals is similar to two previous studies reporting this phenomenon [2, 10]. It has been postulated that remote residuals could be the result of cholesteatoma dispersing during initial surgery [10]. Hypothetically, mucosal traction could also cause migration of cholesteatoma remnants towards the attic and mastoid [37]. The majority of remote pearls (15/24) were found in the pathway of traction from the initial cholesteatoma towards the attic and mastoid. Alternatively, these residuals could reflect incomplete macroscopic identification of residual disease in the first place, emphasising the need for adequate inspection of S2 and A.

### Standardised reporting of residual disease

In the literature reviewed, residuals were described as free text without clear boundaries or categorisation. Information regarding the “difficult access areas” S1 and S2 could not be extracted. Therefore, we advocate the use of STAM areas to report on residual disease. Overlapping areas should be noted specifically, to ensure accurate calculations of residual disease rate per localisation. We urge to register remote residuals as residual cholesteatoma, as these provide valuable insights in to possibly overlooked areas. Knowledge of high-risk areas may encourage surgeons to pay extra attention to these areas in subsequent surgeries.

We also urge uniform reporting on surgery types, for instance by using the SAMEO classification [38]. Length of FU and timing of second-look surgery should be specified, as this is known to influence residual disease rate [6]. Altogether, this will allow for international comparison of reported residual disease rates per localisation, facilitating further research aiming to reduce residual disease after cholesteatoma surgery.

### Limitations, strengths and future perspectives

In our literature review, it is possible relevant articles on residual disease localisation that did not meet our inclusion criteria, were missed. We also acknowledge the limitations posed by the retrospective character of this study.

To the best of our knowledge, this is by far the largest study to date analysing common localisations of residual disease after cholesteatoma surgery. Also, our FU length was longer than the majority of studies reviewed. This study is unique in describing residual disease according to the STAM areas [23], while including the qualitative aspects of residual disease as previously described. In the future, the correlation between localisation of residual disease and patient-related anatomy, such as depth of the sinus tympani, could be analysed.

## Conclusion

In this study, the most common localisation of residual disease following cholesteatoma surgery was the attic, followed by the tympanic cavity. Surgery type is a determining factor not only in residual disease rate, but also in localisation of residual disease. The evident reduction of residual disease following obliteration emphasises the efficacy of this technique, while the overall low rates of residual disease in the mastoid underline the safety of obliteration. We encourage standardised reporting of residuals utilising STAM areas, while specifying overlapping areas and remote residuals. This facilitates comparative research on surgical factors influencing residual disease, while providing useful insights for otologic surgeons.

## Supplementary Information

Below is the link to the electronic supplementary material.

Supplementary table (PDF 119 KB)

Supplementary figure (PDF 119 KB)

## Abbreviations

A: Attic
CWD: Canal wall down procedure
CWD + R: Canal wall down procedure with subsequent reconstruction of the posterior canal wall and obliteration of the epitympanum and mastoid cavity
CWU: Canal wall up procedure
CWUO: Canal wall up procedure with obliteration of the epitympanum and mastoid cavity
FU: Follow-up
M: Mastoid
S1: Anterior epitympanum
S2: Sinus tympani
STP: Subtotal petrosectomy with blind sac closure
T: Tympanic cavity
TCA: Transcanal approach (retro-auricular, endaural and total endoscopic)
