# Supplementary material for: What can we learn from precise reporting of residual disease after various types of cholesteatoma surgery using STAM areas?
Source: Eur Arch Otorhinolaryngol. 2025 Sep 23;282(12):6221–8. doi: 10.1007/s00405-025-09579-3 (PMC12680751; doi:10.1007/s00405-025-09579-3)
Supplement: Supplementary file 1 — Supplementary table (PDF 119 KB) [file 405_2025_9579_MOESM1_ESM.pdf]

Supplementary table 1: Surgery types as classified according to SAMEO[1].

Surgery type	SAMEO[1]
Transcanal procedure: retro-auricular, endaural and total endoscopic (TCA)	S1-2rA1-4MxExOx
Canal wall up procedure (CWU)	S1-2rA4M1a-2bExOx<br>S1-2rA4M1a+2aExOx<br>S1-2rA4M1b+2aExOx
Canal wall up procedure with obliteration of the epitympanum and mastoid cavity (CWUO)	S1-2rA4M1a-2bExO2<br>S1-2rA4M1a+2aExO2<br>S1-2rA4M1b+2aExO2
Canal wall down mastoidectomy (CWD)	S1-2rA4M2cExOx
Canal wall down mastoidectomy with subsequent reconstruction of the posterior canal wall and obliteration of the epitympanum and mastoid cavity (CWD+R)	S1-2rA4M2cE1-2O2
Subtotal petrosectomy with blind sac closure (STP)	S1-2rA4M3a-bExO2

Obliteration of the epitympanum and mastoid was performed using cartilage chips, soft tissue, bone pate and/or hydroxyapatite granules.

1. Yung M, James A, Merkus P, Philips J, Black B, Tono T, Linder T, Dornhoffer J, Incesulu A. International Otology Outcome Group and the International Consensus on the Categorization of Tympanomastoid Surgery. *J Int Adv Otol*. 2018;14(2):216-226. doi:10.5152/iao.2018.5553
