# Supplementary material for: What can we learn from precise reporting of residual disease after various types of cholesteatoma surgery using STAM areas?
Source: Eur Arch Otorhinolaryngol. 2025 Sep 23;282(12):6221–8. doi: 10.1007/s00405-025-09579-3 (PMC12680751; doi:10.1007/s00405-025-09579-3)

Supplementary figure 1:

Localisation of residual cholesteatoma per article included in literature review. In **bold**: residual disease rate in epitympanum (S1 and A), tympanic cavity (T and S2) and mastoid (M), as percentage of all surgeries included. In *italic*: residual disease per localisation as percentage of all residuals. N: number of surgeries, RR: total residual disease rate.

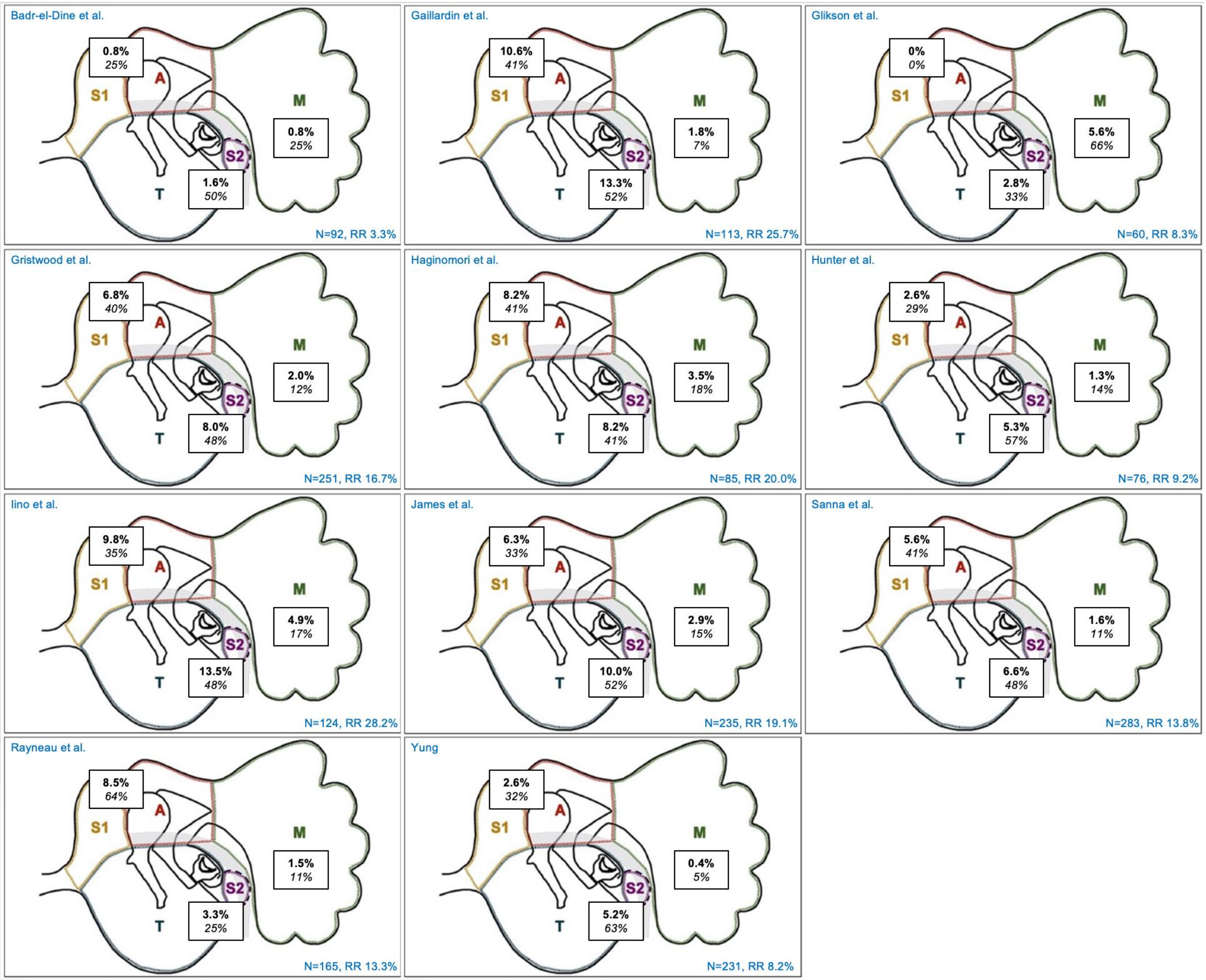

Supplement: Supplementary file 2 — Supplementary figure (PDF 119 KB) [file 405_2025_9579_MOESM2_ESM.pdf]
